# Attention, Visual Perception and their Relationship to Sport Performance in Fencing

**DOI:** 10.2478/hukin-2013-0082

**Published:** 2013-12-31

**Authors:** Mona Mohamed Kamal Hijazi

**Affiliations:** 1 Department of Combats & Aquatics, Faculty of Physical Education, Menoufia University, Egypt.

**Keywords:** Fencing, Visual Discrimination, Visual-Form Constancy, Visual Sequential Memory, Broad External Attentional, Information Processing

## Abstract

Attention and visual perception are important in fencing, as they affect the levels of performance and achievement in fencers. This study identifies the levels of attention and visual perception among male and female fencers and the relationship between attention and visual perception dimensions and the sport performance in fencing. The researcher employed a descriptive method in a sample of 16 fencers during the 2010/2011 season. The sample was comprised of eight males and eight females who participated in the 11-year stage of the Cairo Championships. The Test of Attentional and Interpersonal Style, which was designed by Nideffer and translated by Allawi (1998) was applied. The test consisted of 59 statements that measured seven dimensions. The Test of Visual Perception Skills designed by Alsmadune (2005), which includes seven dimensions was also used. Among females, a positive and statistically significant correlation between the achievement level and Visual Discrimination, Visual-Spatial Relationships, Visual Sequential Memory, Narrow Attentional Focus and Information Processing was observed, while among males, there was a positive and statistically significant correlation between the achievement level and Visual Discrimination, Visual Sequential Memory, Broad External Attentional Focus and Information Processing. For both males and females, a positive and statistically significant correlation between achievement level and Visual Discrimination, Visual Sequential Memory, Broad External Attentional, Narrow Attentional Focus and Information Processing was found. There were statistically significant differences between males and females in Visual Discrimination and Visual-Form Constancy.

## Introduction

Each athletic activity has its own unique psychological characteristics. These characteristics are related to the activity’s natural components and contents, as well as to its requirements for an athlete’s motor abilities, tactical capabilities and higher mental capabilities, such as cognition, perception, memorization, attention and thinking.

According to Deary and Howard (1989), there are performance skills in many sports activities that are difficult to observe. Using film analysis, these authors confirmed eye movements that are invisible to the naked eye. They described the phenomenon as optical anticipation. An example of this phenomenon is related to the difficulty of following a baseball pitch in the last 8–10 feet before it strikes the bat (Deary and Howard, 1989).

Optical anticipation appears more clearly in fencing. The fencer, referee and even viewers can suffer from the phenomenon when they are reviewing and analyzing a filmed performance. Fencing is a sport that is characterized by rapid motor performance. For example, the execution of an attack takes fractions of a second.

The difficulty of reviewing performances in fencing translates to a need for a high degree of optical concentration. Concentration is needed to follow the movements of the feet, body and armed hand of each fencer. A follow-up electrical system is a requirement for this sport.

In fencing, each individual's ability level depends on many variables. Visual variables are the most important, including the accuracy and quality of vision. A visual acuity of 6/6 means that an athlete can see things clearly, but it does not mean that the athlete can determine his/her place in space, how quickly his/her opponent moves or whether the direction of an object will change. Visual processing is responsible for these abilities.

Ariel (2012) suggested that the visual sense plays an important role in physical activity. The visual sense provides athletes with an estimated 80% of the sensory input that occurs during physical activity, especially in activities that require advanced perceptual senses. The perceptual senses are the visual skills that provide athletes with accurate and rapid information; they are considered to be the first step in information processing. The more unclear, incomplete or confused the information and data are, the lower the expected response from the athlete (Ariel, 2012).

Although perception and attention are two separate processes, they are also related. Attention occurs first, but perception interferes with it; attention is a basic condition for perception to occur (Hagemann et al., 2010).

Furthermore, attention and perception are mutually influenced and affected by each other. In many cases, attention can be directed from within an individual, which means that he/she can choose what to focus on or search for specific environmental stimuli to achieve a particular goal (Parkin, 2000).

The direction of attention is usually affected by environmental stimuli located in the individual's area of attention (Hagemann et al., 2010).

Attention is one of the most important mental processes for the growth of an individual's knowledge. Attention enables the individual to select various sensory stimuli to acquire skills and to form appropriate behavioral habits. Attention allows the individual to adapt to his/her environment (Parkin, 2000).

Attention is considered to be one of the important psychological factors that determine superiority in fencing. Attention is of great significance for fencers. Mental abilities, such as attention, perception, intelligence, reaction and expectation, are considered to be the most important factors that must be managed. Mental abilities play a major role in motor behavior, as well as emotions and responses during participation in physical activity in sports. Using mental abilities and emotional factors at their highest limits enhances an athlete’s effort during training and competitions.

For these reasons, the levels of attention and visual perception in fencers were identified and their relationship to a fencer’s achievement level was measured.

### Aims of the study

This study aimed to identify the following parameters:

-Male and female fencers’ attention levels
-Male and female fencers’ visual perception levels
-The relationship between attention and visual perception dimensions and fencers’ achievement levels

## Material and Methods

### Data collection

1. The Test of Attentional and Interpersonal Style (TAIS) was designed by Nideffer and translated by Allawi (1998). The test consisted of 59 statements that measured the following seven dimensions:
  Broad External Attentional Focus (BET), which includes 6 phrases.
  Overloaded by External Stimuli (OET), which includes 12 phrases.
  Broad Internal Attentional Focus (BIT), which includes 8 phrases.
  Overloaded by Internal Stimuli (OIT), which includes 9 phrases.
  Narrow Attentional Focus (NAR), which includes 12 phrases.
  Reduced Attentional Focus (RED), which includes 15 phrases.
  Information Processing (INFP), which includes 19 phrases (Alawi, 1998).
2. The Test of Visual Perception Skills was designed by Alsamadone (2005) and includes seven dimensions, which each consists of 16 phrases.
  - Visual Discrimination (VD).
  - Visual Memory (VM).
  - Visual-Spatial Relationships (VSR).
  - Visual-Form Constancy (VFC).
  - Visual Sequential Memory (VSM).
  - Visual Figure-Ground (VFG).
  - Visual Closure (VC) (Alsamadone, 2005).

### Study method

Taking into account the nature of the study, the researcher used the descriptive method.

### Participants

The study sample included 16 fencers registered in the 2010/2011 season of the Egyptian Federation of Fencing. The sample was comprised of eight males and eight females who participated in the 11-year stage of the Cairo Championships.

### Procedures

#### Survey study

The researcher conducted a survey in a sample of six fencers from the same community, but who were not included in the study population between November 25 and November 30, 2010.

For visual perception items, the validity coefficient ranged from 0.887 to 0.954, and the Alpha reliability coefficient was 0.912.

For attention items, the validity coefficient ranged from 0.895 to 0.931, and the Alpha reliability coefficient was 0.916.

#### Basic study:

The researcher conducted the TAIS and the Test of Visual Perception Skills in the basic study sample at the Egyptian Fencing Club on December 2 – 3, 2010.

## RESULTS

**Figure 1:**
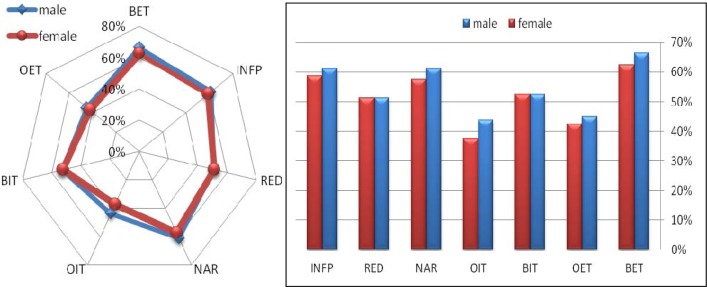
Dimensions of attention for male and female fencers

**Figure 2:**
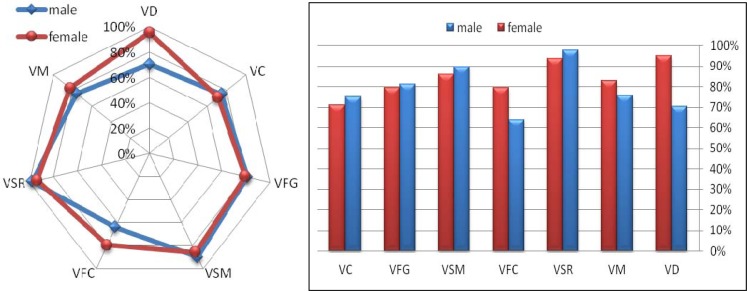
Dimensions of visual perception for male and female fencers

Measurements and analysis showed that the highest attention dimension scores for both males and females were obtained for Broad External Attentional Focus (BET), Information Processing (INFP) and Narrow Attentional Focus (NAR) (Figure 1). Further analysis revealed that the highest visual perception scores for male fencers were obtained for Visual-Spatial Relationships (VSR), Visual Sequential Memory (VSM) and Visual Figure-Ground (VFG); for female fencers, the highest scores were Visual-Spatial Relationships (VSR), Visual Sequential Memory (VSM) and Visual Discrimination (VD) (Figure 2).

For male fencers, the achievement level was correlated with VD and VSM. For female fencers, the achievement level was correlated with VD, VSR and VSM. For the combined group, the achievement level was correlated with VD and VSM (Table 1). Besides, for male fencers, the achievement level was correlated with BET and INFP; For female fencers, the achievement level was correlated with NAR and INFP. For the combined group, the achievement level was correlated with BET, NAR and INFP (Table 2).

Analysis revealed, statistically significant differences between males and females in Visual Discrimination and Visual-Form Constancy (Table 3).

## Discussion

The study sample presented high scores for the following dimensions of attention: BET, INFP and NAR. The dimension BET illustrates that fencers are able to integrate several external variables at the same time. The dimension INFP shows that individuals tend to process a great deal of information, and their informative-cognitive worlds are filled with various types of information. Finally, the dimension NAR expresses fencers’ ability to narrow their attention when desired, and it reflects their ability to focus on one thing or one idea.

There was a lack of significant differences between male and female fencers, and in terms of importance, there was similarity in the attention dimensions between male and female fencers.

There were differences between male and female fencers in terms of the high scores obtained for dimensions of visual perception. Visual Discrimination (VD) was the most important dimension for female fencers, whereas for male fencers, VD occupied the sixth most important dimension.

Furthermore, female fencers were differentiated from male fencers in the Visual-Form Constancy (VFC) dimension. However, there was no clear evidence of significant differences between male and female fencers for the remaining dimensions of visual perception. This finding indicates the distinctiveness of female fencers in some of the visual perception dimensions. However, the entire study sample showed high scores for Visual Memory (VM) and Visual Sequential Memory (VSM).

Athletes require different visual capabilities for different sports. Peripheral vision, optical depth, central vision, visual memory, visual concentration and visual reaction are the most important of these capabilities, and their importance varies according the different requirements of the game in question.

Generally, sports with faster performance requirements, such as fencing, demand advanced visual capabilities and high distinctive visual abilities. Thus, there is a need for athletes to have intact senses.

In the study sample, the attention dimensions that influenced the achievement level were Broad External Attentional Focus (BET), Information Processing (INFP) and Narrow Attentional Focus (NAR). Borysiuk and Waskiewicz (2008) indicated that BET and NAR are interrelated: the focus of optical vision on the detailed optical information is related to the target position and keeping the body in a balanced position (stability). While peripheral vision is responsible for discrimination among stimuli, contrasts, movements and timing (which make up three-dimensional vision), fencers require NAR in the stable state, and during movement, they require peripheral vision. Wood and Abernethy (1997) indicated that the sharpness of vision for moving objects changes from 60% to 70% per second.

Hagemann et al. (2010) suggested that since fencing movements are extremely fast, early recognition of the target area of an opponent's attack is expected to be a key factor for success. This hypothesis was confirmed by the expert-advanced-novice differences observed under all of the experimental conditions. In particular, top-ranked fencers were able to extract markedly more information and use that information to predict the direction of their opponent's attack (Hagemann et al., 2010). Borysiuk and Waskiewicz (2008) suggested that the way information is acquired from the environment and the different perception speeds of individual senses affects the efficacy of technical and tactical actions. The above information is extremely useful in motor training for the development of fencing techniques. A fencer can prepare appropriate strategies to perceive the position of the opponent’s blade - its point and bell guard in particular - to prepare for offensive actions. Knowledge about the opponent’s movements, distance evaluations and visual concentration are the most useful signals. They provide the fencer and the coach with valuable feedback and permit a strategy of switching from focal vision to ambient vision and vice versa to be employed. These strategies develop concentration, which improves the ability to react to initial signals and anticipate the opponent’s actions. They allow a fencer to recognize significant signals and reject misleading information, such as an opponent’s feints (Borysiuk and Waskiewicz, 2008).

The perception dimensions that were clearly related to and affected the achievement level in the study sample were Visual Discrimination (VD), Visual Sequence Memory (VSM) and Visual-Spatial Relationships (VSR). Sight plays a major role in the ability to reach high achievement levels because of its association with attention concentration.

These dimensions are correlated because an increased ability to distinguish optical depth and improve visual concentration and visual memory (visual capabilities) has a positive impact on performance. Zarrad (2001) suggested that these visual capabilities represent an early stage in the preparation for information processing and visual stimuli.

As a knowledge-related ability, visual perception is primarily based on other cognitive abilities that manage visual stimuli, such as attention, memory and thinking. Whereas most of the explanatory cognitive theories regarding visual perception consider it to be more efficient and faster when the optical memory and data storage of stimuli are accurate, we cannot separate visual perception from other cognitive processes, as visual perception overlaps and interacts with other aspects of cognition (Abdul Hamid, 2006).

## Conclusions

Based on results of this study, the following conclusions can be drawn:

1. For fencers, the most important dimensions of attention are Broad External Attentional Focus (BET), Information Processing (INFP) and Narrow Attentional Focus (NAR).
2. The most important dimensions of visual perception for male fencers are Visual-Spatial Relationships (VSR), Visual-Sequential Memory (VSM) and Visual Figure-Ground (VFG); for females, the most important dimensions are Visual-Spatial Relationships (VSR), Visual Sequential Memory (VSM) and Visual Discrimination (VD).
3. There are no significant differences between male and female fencers in the dimensions of attention, but female fencers are differentiated by two dimensions of visual perception: Visual Discrimination (VD) and Visual-Form Constancy (VFC).
4. The most influential correlations between the dimensions of attention and fencer achievement levels are Broad External Attentional Focus (BET), Information Processing (INFP) and Narrow Attentional Focus (NAR).
5. The most influential correlations between the dimensions of visual perception and fencer achievement levels are Visual Sequential Memory (VSM), Visual-Spatial Relationships (VSR) and Visual Discrimination (VD).

## Practical Implications

Based on the aims of the study and the collected data, the following implications can be made:

1. Measures of attention and visual perception should be used in the fencer selection process.
2. When developing technical skills for fencers, variables related to visual perception should be focused on.
3. Internal and external attention should be developed in fencers because of the importance of these abilities in competition.
4. Training programs and skill training should be used to develop dimensions of attention and visual perception according to their relative importance.

## Figures and Tables

**Table 1:** Mean and standard deviation of the achievement level and its correlations with dimensions of visual perception

	achievement level		VD	VM	VSR	VFC	VSM	VFG	VC	SUM
	Mean	ST								
Males	8.000	1.852	^*^0.735	−0.120	0.196	−0.383	^*^0.755	−0.109	−0.181	0.233
Females	6.500	2.449	^*^0.745	0.369	^*^0.750	0.396	^*^0.731	0.611	0.701	^*^0.791
Both	7.250	2.236	^*^0.718	0.077	0.264	0	^*^0.630	0.185	0.159	^*^0.551

* (p < 0.05).

**Table 2:** Correlations between the achievement level and dimensions of attention

	achievement level	BET	OET	BIT	OIT	NAR	RED	INFP
Males	8.000	^*^0.766	−0.126	−0.126	−0.027	0.491	0.500	^*^0.773
Females	6.500	0.414	0.218	0.435	0.436	^*^0.819	−0.311	^*^0.739
Both	7.250	^*^0.511	−0.055	0.174	−0.008	^*^0.510	0.140	^*^0.612

* (p < 0.05).
